# Acute exercise induces distinct quantitative and phenotypical T cell profiles in men with prostate cancer

**DOI:** 10.3389/fspor.2023.1173377

**Published:** 2023-05-30

**Authors:** Erik D. Hanson, Samy Sakkal, Lauren C. Bates-Fraser, Shadney Que, Eunhan Cho, Guillaume Spielmann, Elif Kadife, John A. Violet, Claudio L. Battaglini, Lee Stoner, David B. Bartlett, Glenn K. McConell, Alan Hayes

**Affiliations:** ^1^ Department of Exercise & Sport Science, University of North Carolina, Chapel Hill, NC, United States; ^2^ Lineberger Comprehensive Cancer Center, University of North Carolina, Chapel Hill, NC, United States; ^3^ Human Movement Science Curriculum, University of North Carolina, Chapel Hill, NC, United States; ^4^ Institute for Health and Sport, Victoria University, Melbourne, VIC, Australia; ^5^ School of Kinesiology, Louisiana State University, Baton Rouge, LA, United States; ^6^ Division of Radiation Oncology and Cancer Imaging, Peter MacCallum Cancer Centre, Melbourne, VIC, Australia; ^7^ School of Biosciences and Medicine, Faculty of Health and Medical Sciences, University of Surrey, Guildford, United Kingdom; ^8^ Australian Institute for Musculoskeletal Science (AIMSS), Victoria University, Melbourne, VIC, Australia; ^9^ Department of Medicine—Western Health, Melbourne Medical School, The University of Melbourne, Melbourne, VIC, Australia

**Keywords:** androgen deprivation therapy (ADT), exercise oncology, exercise immunology, conventional t cells (Tconv), unconventional t cells, exercise induced immunosuppression

## Abstract

**Background:**

Reduced testosterone levels can influence immune system function, particularly T cells. Exercise during cancer reduces treatment-related side effects and provide a stimulus to mobilize and redistribute immune cells. However, it is unclear how conventional and unconventional T cells (UTC) respond to acute exercise in prostate cancer survivors compared to healthy controls.

**Methods:**

Age-matched prostate cancer survivors on androgen deprivation therapy (ADT) and those without ADT (PCa) along with non-cancer controls (CON) completed ∼45 min of intermittent cycling with 3 min at 60% of peak power interspersed by 1.5 min of rest. Fresh, unstimulated immune cell populations and intracellular perforin were assessed before (baseline), immediately following (0 h), 2 h, and 24 h post-exercise.

**Results:**

At 0 h, conventional T cell counts increased by 45%–64% with no differences between groups. T cell frequency decreased by −3.5% for CD3^+^ and −4.5% for CD4^+^ cells relative to base at 0 h with CD8^+^ cells experiencing a delayed decrease of −4.5% at 2 h with no group differences. Compared to CON, the frequency of CD8^+^CD57^+^ cells was −18.1% lower in ADT. Despite a potential decrease in maturity, ADT increased CD8^+^perforin^+^ GMFI. CD3^+^Vα7.2^+^CD161^+^ counts, but not frequencies, increased by 69% post-exercise while CD3^+^CD56^+^ cell counts increased by 127% and were preferentially mobilized (+1.7%) immediately following the acute cycling bout. There were no UTC group differences. Cell counts and frequencies returned to baseline by 24 h.

**Conclusion:**

Following acute exercise, prostate cancer survivors demonstrate normal T cell and UTC responses that were comparable to CON. Independent of exercise, ADT is associated with lower CD8^+^ cell maturity (CD57) and perforin frequency that suggests a less mature phenotype. However, higher perforin GMFI may attenuate these changes, with the functional implications of this yet to be determined.

## 1. Introduction

Prostate cancer is one of the most common malignancies in U.S. men (1). With 5-year survival rates of ∼95% in localized disease (1), treatment for prostate cancer is highly effective but is accompanied by many side effects, including alterations in immune cell function (2–9). A robust immune system is critical for immunosurveillance, with regular exercise being one approach to boost immunity (10, 11). Current exercise oncology guidelines exist (12, 13) but do not include recommendations for immune function. This gap is likely the result of a limited number of high-quality studies in these areas.

Prostate cancer treatments for localized disease include prostatectomy, radiation, and hormone therapy. While effective in reducing tumor growth, these treatments include numerous adverse effects. Specifically, androgen deprivation therapy (ADT) has negative impacts on multiple physiological systems (14), with a direct influence on immune function, although the response is complex and paradoxical. Potential benefits of ADT on circulating immune cell function include increased CD3^+^, CD4^+^ and CD8^+^ cell counts (8), greater T cell proliferation (2), and decreased CD4^+^CD25^+^ (Treg) cells (7). Moreover, ADT increases CD3^+^ and CD8^+^ cell infiltration within the prostate (3, 6), although comparable increases in CD4^+^CD25^+^ have also been observed (6). In contrast, others report decreased mitogenic response to stimulation in CD8^+^ cells (5, 7) or only minimal changes in CD3^+^, CD4^+^ and CD8^+^ populations (4, 5), although naïve CD4^+^ T cell frequency was consistently elevated (5, 9). Additionally, increases in pro-inflammatory cytokines have been linked to ADT (15, 16). While greater numbers, proliferation, and infiltration by T cells may improve management of the tumor burden, these benefits may be offset by elevations in chronic inflammation (17) that reduces cellular immunity and leads to a pro-tumor environment (18).

Exercise is a safe, effective, and non-pharmacological means of stimulating the immune system (19–22). Current exercise oncology guidelines recommend bouts of both aerobic and resistance exercise performed at moderate to vigorous intensities for >150 min per week (12, 13). The immune system of healthy individuals is highly responsive to acute exercise via increases in cell number, cytotoxic function, and changes in circulating frequency that reflect redistribution of cells into the tissues (10, 23). However, in cancer survivors, this response has been far less studied (24, 25). In prostate cancer specifically, there are very few acute exercise and immune studies. Initially, modest increases in complete blood counts are reported following resistance exercise (20). Our group observed that one bout of aerobic exercise increased CD3^−^CD56^+^ natural killer (NK) cell frequency in men with PCa (21), although ADT tended to attenuate exercise-induced mobilization compared to non-cancer controls. Additionally, NK cell IFNγ expression was higher with ADT which suggests a more immature phenotype, despite no differences in maturity (CD57^+^) or function (perforin) markers. Recently, high intensity interval exercise increased CD3^−^CD16^+^CD56^+^ NK cells, along with CD3^+^CD8^+^ T cells and CD3^+^CD56^+^ natural killer-like T (NKT-like) cell counts (22). NK and T cell CD57^+^ counts and cytotoxic activity also increased, which appears as a more mature phenotype with higher functional capacity. However, a single bout of acute exercise was insufficient to induce NK cell infiltration within the prostate tissue (19). Collectively, these studies provide an overview of the potential benefits (e.g., increased cell numbers, greater cytotoxicity) of acute exercise during prostate cancer, although NK cells appear overrepresented in oncology populations (24, 26). Consequently, the limited examination of other cell types constitutes a gap in the literature with, to our knowledge, only one study that examines non-NK cell populations in men with prostate cancer (22).

To address this gap, we sought to examine the response of T cell populations to acute exercise in prostate cancer survivors. As part of the adaptive immune system, conventional T cells express a diverse repertoire of α and *β* chains within the T cell receptor (TCR), along with the co-receptors CD4 and CD8 (27). These cells recognize cancer antigens via the major-histocompatibility complex (MHC)−1 (28) and produce cytotoxic proteins (e.g., perforin, granzyme B) and cytokines to enhance the response of other cells. In contrast, unconventional T cells that includes mucosal associated invariant T (MAIT) cells (29) NKT cells (30) that have properties of both the innate and adaptive immune systems (31). MAIT cells are characterized by an invariant Vα7.2 chain within the TCR and are MR-1 restricted (29) while NKT express NK1.1 (CD161) and are restricted via the non-classical MHC-1 protein CD1d (32). MAIT and NKT cells also produce key cytokines and cytotoxic proteins that activate other immune cells and kill tumor cells, respectively (33). While conventional and unconventional T cells are responsive to acute exercise and share functional properties that play critical roles in tumor management (33), these data arise from healthy populations primarily and the response in men with prostate cancer is unknown. Additionally, previous investigations in prostate cancer often lack comparison groups to contextualize the response. We have previously observed impaired stress hormone release (34) and T cell mobilization (35) following acute exercise in breast and prostate cancer survivors, respectively. As such, including non-cancer controls who represent the normal response and separating PCa survivors based on androgen status may help isolate the effects of testosterone suppression on immune function.

Therefore, the purpose of this study was to determine the response of conventional (CD3^+^, CD4^+^ and CD8^+^) and unconventional T cells (MAIT, NKT-like) to acute, moderate intensity aerobic exercise in prostate cancer survivors with and without ADT relative to age-matched controls without cancer. We hypothesized that both conventional and unconventional T cell populations would be mobilized immediately post exercise before returning to resting levels within 24 h of recovery. With the potential of thymic regeneration following prolonged ADT (8), we further hypothesized that men lacking testosterone would exhibit smaller T cell responses to acute exercise and that CD8^+^ T cell and NKT-like T cells would have reduced CD57 and perforin levels.

## 2. Materials and methods

### 2.1. Design

This study was a pre-planned secondary analysis, with the methodology for this study previously published for the primary outcomes (21, 34). In brief, this 4-visit study was completed across 1–2 weeks. Following an initial familiarization session (visit 1), cardiopulmonary exercise testing (CPET, visit 2) was performed. The main testing sessions (visit 3 and 4) were held on subsequent days ∼1 week after visit 2 in the early morning and at the same time of day to minimize the influence of diurnal variations on hormonal concentrations and immune cell circulation in the periphery.

### 2.2. Participants

Men diagnosed with prostate cancer on ADT [ADT; *n* = 11, 67 (2 years)] and not on ADT [PCa; *n* = 14, 67 (2 years)] were recruited from physician collaborators and support groups in Melbourne, Australia along with non-cancer controls [CON; *n* = 8, 64 (3 years)]. ADT and PCa were pathologically diagnosed with prostate cancer via biopsy, were inactive (no regular exercise except for walking for previous 6 months) and were screened for acute or chronic conditions that would contraindicate participation in aerobic exercise. Men on ADT were treated with luteinizing releasing hormone agonists (91%) and anti-androgen receptor (9%) medications for 3+ months prior to enrolling in the study. CON reported no previous history of cancer and met all inclusion and exclusion criteria.

Exclusion criteria included uncontrolled prostate cancer, symptomatic cardiovascular disease, any conditions that caused severe pain with exertion, Type 1 diabetes, uncontrolled Type 2 diabetes, history of bone fractures, inability to engage safely in moderate exercise, or lack of medical clearance from their oncologist, urologist, general practitioner or specialist physician. All participants provided written informed consent. Local ethics committees at Peter MacCallum Cancer Centre, Victoria University, and Western Health approved this project. All procedures were conducted in accordance with principles set out in the Declaration of Helsinki.

### 2.3. Visit 1

To familiarize participants with CPET procedures, participants were fitted with a mask to collect expired gases and to the electronically braked cycle ergometer (Lode, Gronigen, Netherlands). Participants completed 3–4 submaximal stages (0 watts up to 60 or 80 watts). Preassessment guidelines for the main testing session were discussed and included: 2+ hours fasted, no exercise in previous 24 h, and no caffeine or alcohol for previous 12 and 48 h, respectively.

### 2.4. Visit 2

Body composition was determined using dual-energy x-ray absorptiometry (Hologic, Waltham, MA, USA). Quality control checks were completed daily, and all scans were performed and analyzed by the same certified densitometry technician (EH). A CPET was used to determine peak oxygen consumption (VO_2_peak) and exercise trial workloads. Participants completed 1-minute stages with 20 watt increases until volitional exhaustion. Expired gases were sampled every 15 s using automated gas analyzers (Moxus Modular VO_2_ System, AEI Technologies, Pittsburgh, PA, USA) calibrated prior to each test. VO_2_peak was determined as the average oxygen consumption across the last minute of the CPET. Heart rate was assessed continuously via 12 lead electrocardiogram (GE Case Cardiosoft v6.6 ECG Diagnostic Systems, Palatine, IL, USA) and rate of perceived (RPE) exertion using the original Borg scale at the end of each stage.

### 2.5. Visits 3 and 4

Approximately 1 week later, participants arrived in the laboratory between 0600 and 0900 and preassessment guidelines were confirmed verbally. A venous catheter was inserted, and an initial resting blood sample was obtained. Participants then completed an acute, intermittent exercise bout consisting of 10 intervals of 3 min of cycling at 60% of peak wattage from the CPET followed by 1.5 min of passive recovery (45 min total time), as adapted from studies in breast cancer survivors (36). Respiratory gases were sampled throughout the trial and the last minute of each exercise stage was used to determine oxygen consumption, respiratory exchange ratio, and the percentage of exercise relative to VO_2_peak. Heart rate and RPE were obtained in the last 30 s of all stages. Blood samples were also obtained immediately (0 h) and 2 h after exercise. During the recovery period, participants remained seated and consumed water *ad libitum*. Participants went home and returned to the laboratory 24 h after exercise for the last blood sample. They were asked to consume an identical meal prior to visits 3 and 4.

### 2.6. Hematology analysis

Complete blood counts from each time point were determined in duplicate and averaged (Sysmex KX-21N, Kobe, Japan), with a maximal white blood cell difference of 0.1 cells/µl between replicates. Hemoglobin and hematocrit were used to estimate plasma volume shifts following exercise (37).

### 2.7. Peripheral blood mononuclear cells isolation and immunofluorescence

Freshly isolated peripheral blood mononuclear cells were labelled as previously described (38, 39). Briefly, whole blood was diluted in PBS and isolated using SepMate-50 (Stemcell, Vancouver, BC Canada) following manufacturer instructions. Peripheral blood mononuclear cells were washed, counted via hemocytometer and 2 × 10^6^ cells were aliquoted for immunolabelling.

T cell phenotyping was performed via direct immunofluorescence labelling of cells by identifying surface markers with mouse anti-human monoclonal antibodies (Biolegend, San Diego, CA) for 15 min at 4°C in the dark. Lymphocytes were gated using forward and side scatter parameters, with T cells identified as CD3^+^ (APC-Cy7), along with CD4^+^ (V500) and CD8^+^ (PE Cy7). MAIT cells were identified within total T cells using Vα7.2 (PE) and CD161 bright (BV421), along with the CD8^+^ subpopulation, while NKT-like cells were CD3^+^and CD56^+^ (AF647). CD57 (Pacific Blue) was used as a marker of maturity and differentiation status. In addition, intracellular perforin (PE) was assessed as an indicator of function for CD8^+^ and CD3+CD56+ NKT-like cells, as performed previously (21). Briefly, cells were washed twice in PBS before fixation and permeabilization following manufacturer instructions (Cytofix/Cytoperm kit, BD Biosciences, San Jose, CA). Intracellular perforin (PE, Biolegend, San Diego, CA) was stained at 4°C in the dark for 30 min. Cells were then washed and resuspended prior to flow cytometry analysis.

### 2.8. Flow cytometry

**Figure 1:**
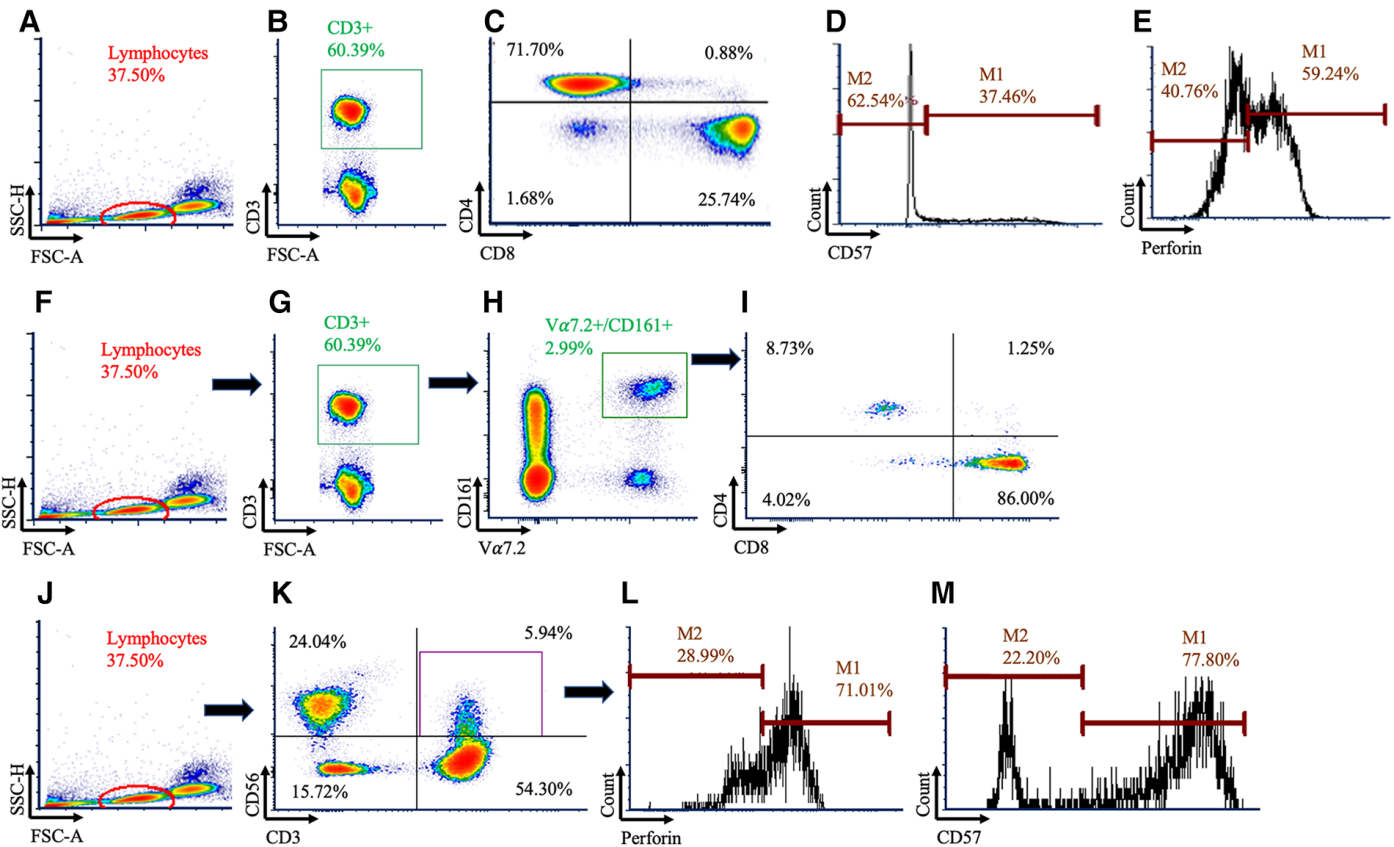
Gating strategy used to identify conventional and unconventional T cells. Conventional T cells were identified using (**A**) lymphocytes, (**B**) followed by CD3^+^ cells (**C**) before being subdivided using CD4^+^ and CD8^+^. Within the CD8^+^ population, histograms were created for (**D**) CD57 and (**E**) perforin. Mucosal Associated Invariant T (MAIT) cells were identified using (**F**) lymphocytes, (**G**) CD3^+^, and (**H**) Vα7.2^+^ and CD161^+^ cells. (**I**) MAIT cells were then subdivided using CD4 and CD8. Natural killer (NK)-like T cells were identified using (**J**) lymphocytes, (**K**) then CD3^+^CD56^+^ cells, and histograms were created for (**L**) perforin and (**M**) CD57.

500,000 events for each sample were acquired and analyzed via flow cytometry on a BD Canto II running FACSDIVA v6.1 (BD Biosciences) software. Flow cytometry analyses were done in duplicate by blinded investigators (EC, GS) using FCS express v7.0 (Pasadena, CA, USA), with the gating strategy shown in Figure 1. Total T cell counts (cells/µl) were determined by multiplying the CD3^+^ cell frequency with the hematology lymphocyte count (38, 39). T cell subpopulation counts were determined by multiplying the T cell count by the frequency of the respective sub-populations. Intracellular perforin expression was quantified as the frequency of cells staining positive along with using the geometric mean fluorescent intensity (GMFI). Fluorescence minus one (FMO) and single color compensation tubes were used with every experiment.

### 2.9. Hormone analysis

Prostate specific antigen (R & D Systems, Minneapolis, MN, USA) and total testosterone levels (Abnova, Taipei City, Taiwan) were determined in duplicate using ELISA, as described previously (34).

### 2.10. Statistical analysis

Group differences for participant characteristics were assessed using one-way ANOVA with Tukey post-hoc analysis. Immune outcomes were analyzed using a linear mixed model, with group and time as fixed factors and subjects as a random effect (21, 33). Group x time interactions were resolved with simple effects examining the group response at each time point. Non-significant interactions were removed from the model. Raw data are presented as mean ± SD, modelled data are presented as mean ± SE with percent changes and model estimates being expressed relative to baseline or the CON group and include 95% confidence intervals. All data were analyzed using Jamovi v2.25 and figures were created in GraphPad Prism version 9 (La Jolla, CA, USA). Statistical significance was set at *P* < 0.05 for main effects and *P* < 0.1 for group x time interactions, given the exploratory nature of this analysis.

## 3. Results

### 3.1. Participants

Men in this study were inactive but relatively healthy otherwise. Men on ADT had higher body mass (*P* = 0.019) and % fat (*P* < 0.001) compared to CON. Apart from total testosterone (*P* < 0.001) and Gleason scores (*P* = 0.007), ADT and PCa were otherwise similar. These data have been published previously (21, 34) and are summarized in Table 1.

**Table 1:** Baseline participant characteristics.

	ADT (*n* = 11)	PCa (*n* = 14)	CON (*n* = 8)
Age (y)	67 ± 7	67 ± 7	64 ± 8
Mass (kg)	91.9 ± 19.8^#^	81.0 ± 8.1	75.1 ± 6.4
Height (cm)	173.1 ± 8.1	174.9 ± 6.0	173.1 ± 3.9
% Fat	29.9 ± 6.7^#^	25.3 ± 3.9	21.0 ± 3.5
Comorbidities^a^	1.5 ± 1.1	1.4 ± 1.1	0.6 ± 1.1
PSA (ng/ml)	9.3 ± 26.8	1.6 ± 2.5	1.9 ± 1.2
Total T (ng/dl)	46.3 ± 23.5^†^^,^^#^	640.7 ± 354.2	669.2 ± 231.1
Gleason Score	8 ± 1^†^	7 ± 1	–
Diagnosis Days	1,241 ± 1,365	1,307 ± 1,099	–
Prostatectomy (%)	36	43	–
Radiation (%)	55	50	–
Length of ADT (d)	596 ± 383	–	–

Mean ± SD.

^a^ Comorbidities is the sum of the following conditions being present: hypertension, hypercholesterolemia, Type II diabetes, smoker, former smoker, and regular alcohol consumption.

^#^ *P* < 0.05 vs. CON.

^†^ *P* < 0.05 vs. PCa.

### 3.2. Physiological response to acute exercise

Absolute VO_2_peak (L/min), maximal HR, and peak power outputs from the CPET were similar between groups with relative VO_2_peak (ml/kg/min) being lower for ADT (*P* = 0.040, Supplementary Table S1) compared to CON. During the intermittent exercise protocol performed at a standardized load of 60% of peak power output, average heart rate and oxygen consumption during exercise were 83.9 ± 10.2% and 80.7 ± 7.8% of maximum with RPE values that were between 12 and 13 and were similar across groups, as reported previously (34).

### 3.3. Complete blood count changes

A group x time interaction was present for leukocyte counts (*P* = 0.016, Supplementary Table S2). There was an initial increase at 0 h, CON increased by 46%, ADT by 57%, and PCa by 39%, with no group difference. At 2 h, leukocytes decreased slightly from 0 h but remained elevated relative to baseline for CON (33%) and ADT (33%) while PCa increased to 46%, which led to a difference in the change from 0 h to 2 h between ADT and PCa (+1.7 × 10^3^ cells/µl; *P* = 0.007). Independent of group, lymphocytes increased by 58% at 0 h before returning to baseline by 2 h and 24 h with similar patterns observed for mixed cells. Neutrophils increased by 46% and 64% at 0 h and 2 h, respectively, but returned to baseline by 24 h. Compared with baseline, plasma volume decreased by −13.4 ± 0.9 (*P* < 0.001) at 0 h, −4.9 ± 1.0 (*P* < 0.001) at 2 h and −2.3 ± 1.0 (*P* = 0.039) at 24 h and were similar across groups.

### 3.4. Conventional T cell counts and frequencies

**Figure 2:**
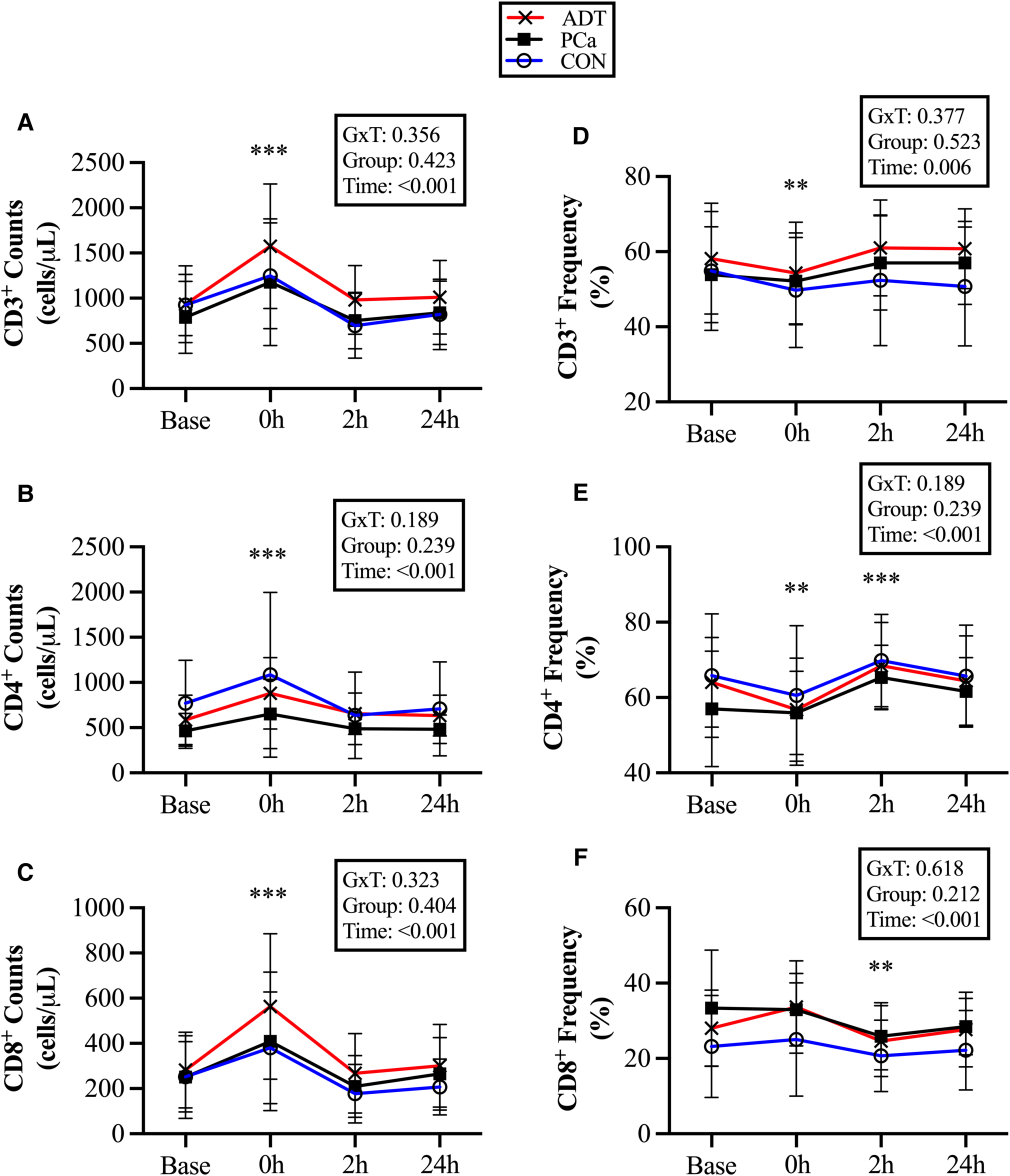
Changes in conventional T cell populations at baseline (base) and during recovery from acute aerobic exercise. Cell counts are reported for (**A**) CD3^+^, (**B**) CD3^+^CD4^+^, and (**C**) CD3^+^CD8^+^ T cells. Cell frequencies reported for (**D**) CD3^+^, (**E**) CD3^+^CD4^+^, and (**F**) CD3^+^CD8^+^ T cells Data are reported as mean and standard deviation. *, **, *** *P* < 0.05, *P* < 0.01, *P* < 0.001 vs. Baseline.

There were no group differences for conventional T cell counts or frequencies. At 0 h, CD3^+^ counts increased by 51% (+457 cells/µl, 95% CI 334, 580; *P* < 0.001, Figure 2A), CD3^+^CD4^+^ counts increased by 45% (+273 cells/µl, 95% CI 183, 364; *P* < 0.001, Figure 2B), and CD3^+^CD8^+^ counts increased by 64% (+167 cells/µl, 95% CI 113, 221; *P* < 0.001, Figure 2C). At 2 h, there was a trend for CD8^+^ counts to decrease below baseline by 19% (−50 cells/µl, 95% CI −105, 4; *P* = 0.074).

At 0 h, CD3 + cell frequency decreased (−3.5%, 95% CI −6.4, −0.7; *P* = 0.018, Figure 2D), while CD3^+^CD4^+^ (−4.5%, 95% CI −7.9, −1.3; *P* = 0.008, Figure 2E) decreased initially but then increased at 2 h (+5.6%, 95% CI 2.3, 8.9; *P* = 0.001). CD3^+^CD8^+^ did not increase at 0 h, but decreased below baseline at 2 h (−4.5%, 95% CI −7.6, −1.5, *P* = 0.005, Figure 2F).

### 3.5. Cytotoxic T cell CD57 and perforin counts, frequencies, and expression levels

CD57 was used as a marker of maturity within CD3^+^CD8^+^ cells. At 0 h, CD3^+^CD8^+^CD57^+^ counts increased by 75% (+79 cells/µl, 95% CI 31, 127; *P* = 0.002, Table 2) with no change in frequency over time. ADT had a lower frequency of CD3^+^CD8^+^CD57^+^ cells compared to both PCa (−15.1%, 95% CI −22.5, −7.8; *P* < 0.001) and CON (−18.1%, 95% CI −26.3, −9.9; *P* < 0.001).

**Table 2:** CD57 and perforin expression in CD3^+^CD8^+^ T cells before and after acute exercise.

		Base	0 h	2 h	24 h
CD8^+^CD57^+^ (×10^3^ cells/µl)	Total	105 ± 23	184 ± 24**	83 ± 24	111 ± 23
	ADT	92 ± 36	204 ± 43	90 ± 41	56 ± 38
	PCa	110 ± 38	150 ± 38	68 ± 38	111 ± 38
	CON	113 ± 46	199 ± 46	93 ± 46	166 ± 46
CD8^+^CD57^+^ (%)	Total	42.2 ± 3.2	35.6 ± 3.4	46.3 ± 3.1	42.0 ± 3.3
	ADT	34.3 ± 5.1^#^^,^^†^	26.6 ± 6.3^#^^,^^†^	33.8 ± 5.3 ^#^^,^^†^	27.0 ± 5.1 ^#^^,^^†^
	PCa	47.6 ± 5.3	44.1 ± 5.1	42.4 ± 5.1	48.2 ± 5.1
	CON	44.7 ± 6.3	36.1 ± 6.3	62.8 ± 5.9	50.7 ± 6.9
CD8^+^Perf^+^ (×10^3^ cells/µl)	Total	105 ± 23	184 ± 24^**^	83 ± 24^*^	111 ± 23
	ADT	92 ± 36	204 ± 43	90 ± 41	56 ± 38
	PCa	110 ± 38	150 ± 38	68 ± 38	111 ± 38
	CON	113 ± 46	199 ± 46	93 ± 46	166 ± 46
CD8^+^Perf^+^ (%)	Total	71.2 ± 4.0	56.7 ± 4.3	58.7 ± 3.9	52.9 ± 3.9
	ADT	62.5 ± 6.3	61.6 ± 7.9	39.0 ± 6.1^*^	49.4 ± 6.1
	PCa	76.3 ± 6.3	60.1 ± 6.3	59.5 ± 6.1	60.6 ± 6.1
	CON	74.7 ± 7.9	48.4 ± 7.9*	77.7 ± 7.4	48.8 ± 7.7*
CD8^+^Perf^+^ (GMFI)	Total	2,398 ± 439	3,167 ± 464	3,259 ± 433	3,094 ± 433
	ADT	3,918 ± 688 ^#^^,^^†^	4,868 ± 848 ^#^^,^^†^	3,735 ± 688 ^#^^,^^†^	5,633 ± 688 ^#^^,^^†^
	PCa	1,871 ± 718	2,348 ± 688	3,955 ± 688	2,578 ± 688
	CON	1,404 ± 862	2,286 ± 862	2,086 ± 862	1,073 ± 862

Mean ± SE from the estimated marginal mean. When statistical significance is indicated on the Total, this represents a main effect. GMFI, geometric mean fluorescent intensity.

* *P* < 0.05

** *P* < 0.01 vs. baseline.

^#^ *P* < 0.05 vs. CON.

^†^ *P* < 0.05 vs. PCa.

At 0 h, CD3^+^CD8^+^Perforin^+^ counts increased by 68% (+120 cells/µl, 95% CI 60, 181; *P* < 0.001, Table 2) before decreasing below baseline at 2 h (−68 cells/µl, 95% CI −127, −10; *P* = 0.026). For CD3^+^CD8^+^Perforin^+^ frequency, there was a group x time interaction (*P* = 0.031). There were no baseline differences. At 0 h, both CON (−26.3%, 95% CI −29.0, −23.6; *P* = 0.018) and PCa decreased (−16.2%, 95% CI −17.0, −15.4; *p* = 0.074), although the latter demonstrated only a trend. ADT was unchanged initially but then decreased below baseline at 2 h (−23.6%, 95% CI 20.9, −26.3, −20.9; *P* = 0.008). For GMFI, ADT CD3^+^CD8^+^Perforin^+^ expression was higher than both PCa (+1,851, 95% CI 614, 3,088; *P* = 0.007) and Con (+2,826, 95% CI 1,435, 4,218; *P* < 0.001).

### 3.6. Unconventional T cell counts and frequencies

**Figure 3:**
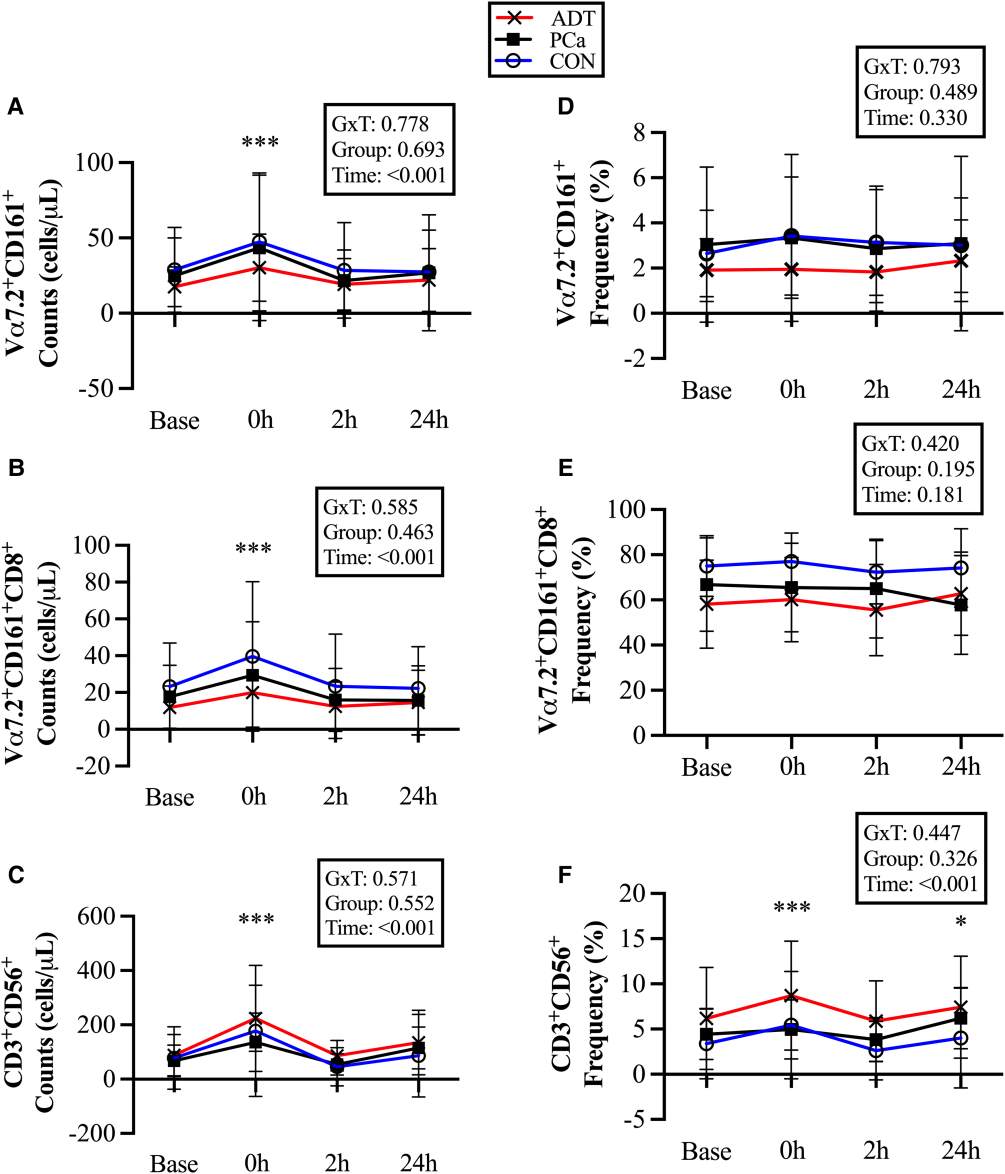
Changes in unconventional T cell populations at baseline (base) and during recovery from acute aerobic exercise. Cell counts are reported for (**A**) CD3^+^Vα7.2^+^CD161^+^ Mucosal Associated Invariant T (MAIT) cells, (**B**) CD3^+^Vα7.2^+^CD161^+^CD8^+^ MAIT cells, and (**C**) CD3^+^CD56^+^ Natural killer (NK)-like T cells. Cell frequencies are reported for (**D**) CD3^+^Vα7.2^+^CD161^+^ MAIT cells, (**E**) CD3^+^Vα7.2^+^CD161^+^CD8^+^ MAIT cells, and (**F**) CD3^+^CD56^+^ NKT-like cells. Data are reported as mean and standard deviation. *, **, *** *P* < 0.05, *P* < 0.01, *P* < 0.001 vs. Baseline.

At 0 h, CD3^+^Vα7.2^+^CD161^+^ counts increased by 69% (+17 cells/µl, 95% CI 9, 24; *P* < 0.001, Figure 3A), CD3^+^Vα7.2^+^CD161^+^CD8^+^ counts increased by 69% (+12 cells/µl, 95% CI 7, 17; *P* < 0.001, Figure 3B), and CD3^+^CD56^+^counts increased by 127% (+99 cells/µl, 95% CI 68, 131; *P* < 0.001, Figure 3C).

There were no changes in CD3^+^Vα7.2^+^CD161^+^ or CD3^+^Vα7.2^+^CD161^+^CD8^+^ cell frequency (Figures 3D & 3E), while CD3^+^CD56^+^cells were increased at 0 h (+1.7%, 95% CI 0.8, 2.6; *P* < 0.001, Figure 3F) and 24 h (+1.1%, 95% CI 0.2, 2.1; *P* = 0.017).

### 3.7. NKT-like cell CD57 and perforin counts, frequencies, and expression levels

At 0 h, CD3^+^CD56^+^CD57^+^ counts increased by 138% (+65 cells/µl, 95% CI 43, 88; *P* < 0.001, Table 3) with a non-significant increase in frequency at 0 h followed by a decrease at 2 h (−3.6% 95% CI −9.3, −1.7; *P* = 0.006) compared to baseline.

**Table 3:** CD57 and perforin expression in CD3^+^CD56^+^ T cells before and after acute.

		Base	0 h	2 h	24 h
CD3^+^CD56^+^CD57^+^ (×10^3^ cells/µl)	Total	47 ± 15	112 ± 15***	32 ± 15	62 ± 16
	ADT	48 ± 24	126 ± 24	39 ± 24	64 ± 24
	PCa	38 ± 25	80 ± 25	27 ± 25	54 ± 27
	CON	56 ± 30	131 ± 30	30 ± 30	68 ± 31
CD3^+^CD56^+^CD57^+^ (%)	Total	56.6 ± 4.4	59.5 ± 4.4	51.1 ± 4.5**	52.9 ± 4.5
	ADT	52.0 ± 6.8	58.6 ± 6.8	49.3 ± 6.9	46.8 ± 6.8
	PCa	52.5 ± 7.6	55.1 ± 7.6	47.5 ± 7.6	52.8 ± 7.7
	CON	65.3 ± 8.6	64.9 ± 8.6	56.4 ± 8.6	59.2 ± 8.7
CD3^+^CD56^+^Perf^+^ (×10^3^ cells/µl)	Total	41 ± 14	93 ± 14	29 ± 14	55 ± 14
	ADT	59 ± 21	161 ± 21***	55 ± 22	80 ± 22
	PCa	38 ± 23	69 ± 23	21 ± 23	52 ± 25
	CON	26 ± 27	50 ± 27	11 ± 27	32 ± 28
CD3^+^CD56^+^Perf^+^ (%)	Total	51.3 ± 5.7	57.2 ± 5.7	46.7 ± 5.7	47.6 ± 5.8
	ADT	50.8 ± 8.8	62.2 ± 8.8	51.5 ± 8.9	49.5 ± 8.8
	PCa	52.3 ± 9.7	54.3 ± 9.7	42.2 ± 9.7	48.9 ± 10.0
	CON	50.7 ± 11.0	55.0 ± 11.0	46.3 ± 11.0	44.5 ± 11.2
CD3^+^CD56^+^Perf^+^ (GMFI)	Total	4,587 ± 517	4,915 ± 512	4,643 ± 520	4,908 ± 525
	ADT	5,012 ± 786	5,281 ± 786	5,268 ± 803	5,664 ± 786
	PCa	4,246 ± 850	4,573 ± 824	4,202 ± 850	4,152 ± 906
	CON	4,504 ± 1,032	4,892 ± 1,032	4,460 ± 1,032	4,908 ± 1,022

Mean ± SE from the estimated marginal mean. When statistical significance is indicated on the Total, this represents a main effect.

** *P* < 0.01

*** *P* < 0.001 vs. baseline.

There was a group x time interaction for CD3^+^CD56^+^Perforin^+^ counts, with ADT increasing by 172% at 0 h (+101 cell/uL, 95% CI 60, 142, *P* < 0.001, Table 3) while PCa (+82%, *P* = 0.115) and CON (+92%, *P* = 0.295) experienced smaller, non-significant rises. CD3^+^CD56^+^Perforin^+^ frequency revealed an overall effect of time (*P* = 0.025). However, *post hoc* analysis revealed that none of the time points were different from baseline. Perforin GMFI was unchanged across group or time.

## 4. Discussion

ADT slows tumor progression and increases survival but has many potential side effects, including alterations in the immune system. Regular exercise targets many adverse outcomes, but specifics regarding best practice on enhancing immune function in cancer survivors remains unclear. In the present study, the effects of acute exercise on T cell counts, frequency, and functional markers in men with prostate cancer were examined. We found that (i) the conventional T cell response in prostate cancer survivors is similar to non-cancer controls; (ii) overall CD57 frequency in CD8^+^ T cells is lower with ADT, suggesting reduced maturation in the absence of testosterone, yet higher perforin expression levels were observed that may represent a compensatory response; and (iii) UTCs demonstrate increased counts with acute exercise in all groups, with NKT-like cells showing preferential mobilization whereas MAIT cells did not. Collectively, these data provide evidence that moderate-vigorous bouts of acute exercise effectively mobilize T cells during PCa, with apparent recovery within 24 h without reduced immune cell counts, frequency, or potential function.

To provide context, the limitations and strengths of the study are presented initially. Limitations include modest sample sizes per group and variable time since diagnosis. Both may have impacted the ability to detect group x time interactions, which is why a value of *P* < 0.1 was used. No direct measurements of cytotoxic function were assessed. Instead, intracellular perforin levels were used as a surrogate measure of cellular function. Finally, NKT-like cells (vs. bona fide NKT cell) were examined, as access to the Cd1D tetramer was not readily available at study inception. The use of NKT-like cells is a common limitation in exercise immunology (33), and likely contributes to the higher-than-expected frequencies observed compared to actual NKT cells (31). Strengths of the study include reporting of all immune outcomes for pooling of data in future analyses (24), examining PCa and ADT separately to better isolate exercise effects, the use of multiple recovery time points, and the inclusion of more complex phenotyping and investigating UTCs.

In men with prostate cancer, acute exercise produces a typical mobilization of conventional T cells that mirrors that of non-cancer controls. All T cell counts increased at 0 h, with greater relative increases in the CD8^+^ populations. While CD3^+^ cell counts increased, the frequency decreased overall as concomitant increases in NK cells occurred at greater rates in these men (21). Additionally, CD8^+^ T cell frequency increased while CD4^+^ frequency decreased, with the relative changes of these sub-populations mirroring each other here and elsewhere (35, 39). Overall, these T cell count and frequency changes are consistent with previous work in breast cancer survivors (35) while supporting and extending recent work in prostate cancer survivors (22). A shift towards cells with greater cytotoxicity following adrenergic receptor stimulation is well established, as NK and CD8+ cells demonstrate greater mobilization following acute exercise, psychological stress, and β-agonist infusion (40). As we previously reported that men with prostate cancer have attenuated epinephrine responses to acute exercise (34), it interesting that neither T cells (current study) or NK cell mobilization (21) were adversely impacted. This suggests that other factors may compensate for the reduced epinephrine response, such as changes in shear stress or myokine release (41).

While conventional T cell mobilization appears to be unimpaired after acute exercise, alterations in CD57 and perforin may have functional implications. We report an increase in CD8^+^CD57^+^ counts post-exercise, which is consistent with previous work in healthy men (42) as acute exercise generally increases mobilization of senescent (CD28^−^CD57^+^) T cells (43). In contrast, the frequency of CD8^+^CD57^+^ cells was unchanged, with a non-significant 6.6% decrease at 0H. The lack of change is similar to our work in NK cells (21), but contrasts recent work using HIIT which found increased CD57 frequency after a graded exercise test (22). Differences in exercise intensity (60% vs. 100% of peak power output in the HIIT study) may provide an explanation. Perhaps more interesting is that ADT decreased the frequency of CD8^+^CD57^+^ cells compared to both PCa and CON, an effect that was independent of exercise, although the total counts were not impacted that suggests no lack of availability of these cells. One interpretation is that castrate levels of testosterone lead to less mature CD8^+^ T cells, which aligns with our hypothesis. Reports of higher rates of T cell proliferation (2), decreased response to simulation (7) and increases in naïve CD8+ T cells (8) indirectly support this finding. Changes in thymus mass and output may be a potential mechanism explaining the reductions in mature T cells with ADT. Thymic involution occurs with age (44) and increased testosterone levels (45), but can be reversed following castration (8). ADT increased naïve CD4^+^ T (but not CD8^+^) cells (5), that supports increased thymic output of less mature cells. However, CD57 was not included in the analysis and the present study did not include CD4^+^CD57^+^ cells, so direct comparisons are limited.

In support of ADT leading to less mature CD8^+^ cells, perforin frequency was ∼12%–14% lower at baseline. However, the group main effect revealing potential differences between ADT and the other groups was not examined because of the significant group x time interaction. Instead, ADT appeared to delay the decrease in perforin frequency until 2H, whereas CON and PCa both were reduced at 0H, with the reasons behind this currently being unclear. Conversely, perforin GMFI during ADT was increased within the CD8^+^ cells. A decrease in maturity status (as determined by CD57%) was not anticipated to augment cytotoxic protein expression, as men with prostate cancer report CD56^bright^ NK cells have substantially lower perforin MFI and CD57 expression compared to CD56^dim^ (21). Alternatively, higher perforin expression may be a compensatory shift within these cells. As cytotoxic or stimulation assays were not performed, the functional implications of this remain unknown.

To our knowledge, this is the first instance where the MAIT cell response to acute exercise has been quantified in prostate cancer survivors. There was a 69% increase in counts at 0 h, which falls between the 46% increase reported in breast cancer survivors but less than the 137% of non-cancer controls performing an identical bout of acute exercise (35). Somewhat surprisingly, acute exercise did not preferentially mobilize MAIT cells within the T cell pool. This is contrary to our prior work in healthy and breast cancer populations (35, 38, 39), which report consistent increases of 0.7%–1.7% that occur in an exercise intensity-dependent manner. As the intensity of our intermittent exercise protocol aligns well with other submaximal bouts (35, 38), this explanation seems unlikely. Alternatively, the proportions of CD8^+^ MAIT cells was lower (10%–15%) compared to younger men (38, 39). However, this MAIT cell sub-population is less responsive to acute exercise, thereby a lower frequency of these cells argues for (not against) preferential mobilization. Prostate cancer treatment does not appear to alter MAIT cell numbers but decreases proliferation and immune cell IFNγ production (4). However, it is not apparent if this is the direct result of testosterone suppression, as only 67% of patients were on ADT. While not assessed in the current study, acute and chronic exercise have potential to offset ADT-related impairments in MAIT cell cytokine production. For example, TNFα production was greater (with a trend towards increased IFNγ) in healthy young men and older women immediately following a single exercise bout (35, 38). Women with breast cancer improved stimulated TNFα and IFNγ following a 16 week exercise intervention (35), which suggests that training status influences this response.

In NKT-like (CD3^+^CD56^+^) cells, cell counts and frequencies both increased post-exercise. Cell counts for NKT-like cells expressing CD57 and perforin both increased at 0H with only minor changes in frequency. The increased counts were similar to studies in healthy individuals (46) but reduced compared to maximal exercise (22). Together, this indicates there may be an intensity threshold that leads to increased CD57 and Granzyme B frequency (22) that were not observed in our study. With limited work to date, exercise intensity differences seem plausible but need to be investigated within the same study. The mobilization of more mature NKT-like cells with greater cytotoxic protein frequency seems promising, particularly as NKT cells are a target for immunotherapy (47). However, the lack of functional assays using these cells from cancer survivors and the use of CD1d to distinguish classical NKT cells (vs. NKT-like cells) during acute and chronic exercise currently limits our knowledge in this area.

From the findings that the acute exercise response in men with prostate cancer is normal, three implications for consideration regarding this type of physical activity are presented. (i) While a single exercise bout was insufficient (19, 48), multiple sessions enhanced immune cell infiltration of prostate tumors (49) that aligns with data from pre-clinical models (50, 51). (ii) Breast cancer cell culture experiments reveal that acute but not chronic exercise reduce cell viability (52), leading to the hypothesis that “every exercise bout matters” (53). Together, these may be potential mechanisms by which increasing amounts of physical activity reduce prostate cancer specific mortality (54). (iii) Finally, the exercise immune landscape is becoming increasing complex, as more cells are included with greater phenotypic complexity. This reinforces the need to have standardized techniques and outcome reporting (24) such that additional meta-analyses can be performed to better isolate the effects of exercise mode or specific cell types on immune function in cancer survivors (26).

In summary, conventional and unconventional T cell numbers and frequencies in prostate cancer survivors demonstrate a normal response to moderate-vigorous acute exercise, which is consistent with our prior NK cell work. MAIT cells do not undergo preferential mobilization post exercise, with this response in older men conflicting with previous findings. Independent of exercise, ADT had lower CD8^+^ cell maturity (CD57) and perforin frequency that suggests a less mature phenotype than CON that appears to be influenced by testosterone levels. However, higher perforin MFI may attenuate these changes, although the functional implications of this is yet to be determined. Finally, UTC/T cell responses had normalized by 24H. This implies that consecutive training sessions can be performed with minimal concerns of adverse immune system effects during prostate cancer. However, the response following multiple consecutive bouts of acute exercise needs to be determined to test this hypothesis.

## Data Availability

The raw data supporting the conclusions of this article will be made available by the authors, without undue reservation.
