# Supplementary material for: Acute exercise induces distinct quantitative and phenotypical T cell profiles in men with prostate cancer
Source: Front Sports Act Living. 2023 May 30;5:1173377. doi: 10.3389/fspor.2023.1173377 (PMC10266416; doi:10.3389/fspor.2023.1173377)
Supplement: Supplementary file 1 [file Table1.docx]

| **Supplemental Table 1.** Physiological response to the maximal cardiopulmonary exercise test and the intermittent exercise protocol. | | | |
| --- | --- | --- | --- |
|  | **ADT** | **PCa** | **CON** |
| **CPET** |  |  |  |
| VO_2_peak (l/min) | 2.1 ± 0.6 | 2.0 ± 0.6 | 2.4 ± 0.6 |
| VO_2_peak (ml/kg/min) | 23.9 ± 8.4 # | 25.1 ± 5.8 | 32.4 ± 8.1 |
| Maximal HR (bpm) | 157 ± 25 | 152 ± 13 | 158 ± 11 |
| Peak Power Output (w) | 175 ± 72 | 166 ± 58 | 206 ± 40 |
|  |  |  |  |
| **Intermittent Exercise Protocol** |  |  |  |
| Trial workload (w) | 107 ± 43 | 96 ± 31 | 123 ± 26 |
| Exercise HR (bpm) | 131 ± 20 | 124 ± 16 | 130 ± 10 |
| Exercise % HRmax | 85.1 ± 15.1 | 82.1 ± 9.5 | 82.1 ± 9.5 |
| Exercise VO_2_ (ml/kg/min) | 20.7 ± 5.6 | 19.9 ± 3.8 | 24.7 ± 4.5 |
| Exercise % VO_2_peak | 84.8 ± 8.1 | 80.5 ± 9.0 | 77.6 ± 6.4 |
| Exercise RPE | 12.2 ± 2.3 | 12.1 ± 1.3 | 13.0 ± 2.2 |
| Recovery HR (bpm) | 104 ± 18 | 101 ± 15 | 101 ± 15 |
| Recovery % HRmax | 67.9 ± 14.9 | 67.1 ± 10.4 | 64.7 ± 4.4 |
| Recovery VO_2_ (ml/kg/min) | 10.8 ± 1.6 | 11.1 ± 1.9 | 12.5 ± 1.8 |
| Recovery % VO_2_ | 41.5 ± 16.9 | 46.1 ± 10.3 | 39.9 ± 7.3 |
| Mean ± SD. CPET = cardiopulmonary exercise test; VO_2_peak = peak oxygen consumption; bpm = beats per minute; w = watts; HR = heart rate; RPE = rate of perceived exertion; RER = respiratory exchange ratio; PV = plasma volume  # P<0.05 vs. CON | | | |
