# Supplementary material for: Acute exercise induces distinct quantitative and phenotypical T cell profiles in men with prostate cancer
Source: Front Sports Act Living. 2023 May 30;5:1173377. doi: 10.3389/fspor.2023.1173377 (PMC10266416; doi:10.3389/fspor.2023.1173377)
Supplement: Supplementary file 2 [file Table2.docx]

| **Supplemental Table 2.**  Complete blood counts and plasma volume (PV) shifts before and after acute exercise. | | | | | |
| --- | --- | --- | --- | --- | --- |
|  |  | **Base** | **0h** | **2h** | **24h** |
| Leukocytes | Total | 5.3 ± 0.2 | 7.9 ± 0.2 | 7.3 ± 0.2 | 5.4 ± 0.2 |
| (x10^3^ cells/µL) | ADT | 5.5 ± 0.4 | 8.5 ± 0.4*** | 7.2 ± 0.4*** | 5.3 ± 0.4 |
|  | PCa | 5.1 ± 0.4 | 7.1 ± 0.4*** | 7.5 ± 0.4*** | 5.4 ± 0.4 |
|  | CON | 5.4 ± 0.5 | 7.9 ± 0.5*** | 7.2 ± 0.5*** | 5.3 ± 0.5 |
| Lymphocytes | Total | 1.6 ± 0.1 | 2.5 ± 0.1*** | 1.5 ± 0.1 | 1.6 ± 0.1 |
| (x10^3^ cells/µL) | ADT | 1.6 ± 0.2 | 2.8 ± 0.2 | 1.6 ± 0.2 | 1.6 ± 0.2 |
|  | PCa | 1.5 ± 0.2 | 2.3 ± 0.2 | 1.4 ± 0.2 | 1.5 ± 0.2 |
|  | CON | 1.8 ± 0.2 | 2.5 ± 0.2 | 1.4 ± 0.2 | 1.7 ± 0.2 |
| Mixed | Total | 0.6 ± 0.1 | 0.7 ± 0.1*** | 0.6 ± 0.1 | 0.6 ± 0.1 |
| (x10^3^ cells/µL) | ADT | 0.5 ± 0.1 | 0.7 ± 0.1 | 0.5 ± 0.1 | 0.5 ± 0.1 |
|  | PCa | 0.6 ± 0.1 | 0.7 ± 0.1 | 0.6 ± 0.1 | 0.6 ± 0.1 |
|  | CON | 0.5 ± 0.1 | 0.8 ± 0.1 | 0.6 ± 0.1 | 0.6 ± 0.1 |
| Neutrophils | Total | 3.2 ± 0.2 | 4.7 ± 0.2*** | 5.3 ± 0.2*** | 3.2 ± 0.2 |
| (x10^3^ cells/µL) | ADT | 3.1 ± 0.3 | 4.2 ± 0.3 | 5.5 ± 0.3 | 3.3 ± 0.3 |
|  | PCa | 3.2 ± 0.4 | 4.8 ± 0.4 | 5.3 ± 0.4 | 3.2 ± 0.4 |
|  | CON | 3.5 ± 0.4 | 5.2 ± 0.4 | 5.2 ± 0.4 | 3.2 ± 0.4 |
| PV Shift | Total | - | -13.4 ± 0.9 *** | -4.9 ± 1.0 *** | -2.3 ± 1.0 * |
| (%) | ADT | - | -13.7 ± 1.5 | -4.6 ± 1.5 | 0.1 ± 1.6 |
|  | PCa | - | -12.7 ± 1.5 | -6.6 ± 1.6 | -3.9 ± 1.5 |
|  | CON | - | -13.9 ± 1.8 | -3.3 ± 1.8 | -3.3 ± 1.8 |
| Mean ± SE from the estimated marginal mean. When statistical significance is indicated on the Total, this represents a main effect. ADT = androgen deprivation therapy; PCa = prostate cancer; CON = controls  * P<0.05 *** P<0.001 vs. baseline | | | | | |
